# The emerging role of PET imaging in dementia

**DOI:** 10.12688/f1000research.11603.1

**Published:** 2017-10-12

**Authors:** Leonardo Iaccarino, Arianna Sala, Silvia Paola Caminiti, Daniela Perani

**Affiliations:** 1 Vita-Salute San Raffaele University, Milan, Italy; 2 In Vivo Human Molecular and Structural Neuroimaging Unit, Division of Neuroscience, IRCCS San Raffaele Scientific Institute, Milan, Italy; 3 Nuclear Medicine Unit, IRCCS San Raffaele Hospital, Milan, Italy

**Keywords:** fluorodeoxyglucose, amyloid, tau, neuroinflammation, molecular imaging, diagnosis, prognosis, biomarker, dementia, PET

## Abstract

A compelling need in the field of neurodegenerative diseases is the development and validation of biomarkers for early identification and differential diagnosis. The availability of positron emission tomography (PET) neuroimaging tools for the assessment of molecular biology and neuropathology has opened new venues in the diagnostic design and the conduction of new clinical trials. PET techniques, allowing the in vivo assessment of brain function and pathology changes, are increasingly showing great potential in supporting clinical diagnosis also in the early and even preclinical phases of dementia. This review will summarize the most recent evidence on fluorine-18 fluorodeoxyglucose-, amyloid -, tau -, and neuroinflammation - PET tools, highlighting strengths and limitations and possible new perspectives in research and clinical applications. Appropriate use of PET tools is crucial for a prompt diagnosis and target evaluation of new developed drugs aimed at slowing or preventing dementia.

## Introduction

The last decades have progressively witnessed a shift from a solely clinical diagnosis to a biomarker-supported diagnosis, and molecular neuroimaging techniques such as positron emission tomography (PET) have played a leading role in the dementia diagnostic work-up ^1– 8^. PET techniques have provided major advances, promoting novel approaches to support an early and differential dementia diagnosis. An accurate quantification, together with *ad hoc* PET techniques, can nowadays detect very subtle, but significant, pathological and functional neuronal changes, even before clinical symptoms arise. This is crucial for early interventions, personalized care planning, and inclusion in clinical trials ^9^. Of note, several studies have demonstrated that PET techniques fully show their diagnostic and prognostic value especially when appropriate quantification methods are applied ^10– 13^. These cardinal issues include timing and protocol of acquisition, parametric modelling and estimation, and the critical definition of the reference region to be used for semi-quantification.

Here, we review the most recent advances, strengths, and weaknesses of four of the leading or novel PET tools (or both) in the dementia research field, namely ^18^F-FDG (fluorine-18 fluorodeoxyglucose), amyloid, tau, and neuroinflammation PET imaging. The progressive implementation of these techniques, together with the standardization of appropriate methodologies, will allow unique breakthroughs in our understanding of neurodegeneration and will have remarkable implications for diagnostic algorithms and therapy monitoring.

## ^18^F-fluorodeoxyglucose PET

The fundamentals of ^18^F-FDG PET are well established and are based on extensively explored molecular mechanisms ^9^. ^18^F-FDG PET signal reflects astrocyte/neuron coupled energy consumption ^14– 17^, and pioneering and recent studies support the notion that astrocytes play a central role in neuronal glucose consumption ^18^. Decrease of ^18^F-FDG PET uptake is considered to be a direct index of synaptic dysfunction, which can result from a variety of neuropathological events, including but not limited to altered intracellular signaling cascades and mitochondria bioenergetics, impaired neurotransmitter release, and accumulation of neurotoxic protein species ^9, 19^. Of note, ^18^F-FDG PET has been shown to be extremely sensitive to any perturbation in glucose metabolism, being able to capture neurodegeneration not only due to local pathological and biochemical alterations but also due to long-distance functional deafferentations ^9^.

With regard to neurodegenerative diseases, a large and growing body of research has provided convincing and consistent evidence for highly specific patterns of ^18^F-FDG PET hypometabolism in distinct dementia conditions ^9, 20– 26^, even before manifest brain atrophy occurs ^27, 28^. ^18^F-FDG PET can provide support to differential diagnosis based on disease-specific hypometabolism patterns ^20, 23, 25, 26, 29, 30^. The ability of ^18^F-FDG PET to capture disease-specific patterns led to the inclusion of ^18^F-FDG PET hypometabolism as a supportive feature in the clinical/research diagnostic criteria of multiple dementia conditions ^1– 6, 8^. ^18^F-FDG PET is an accurate tool for early detection and estimation of increased risk for future dementia conversion ^31^ in the earliest clinical phases of disease, such as in subjects with mild cognitive impairment (MCI), as well as in preclinical cases (asymptomatic subjects at risk or asymptomatic carriers of pathogenetic mutations) ^25, 32, 33^, providing highly relevant prognostic information for clinical use ^34^. Crucially, the ability of ^18^F-FDG PET to identify heterogeneous hypometabolism patterns in MCI allows clinicians to predict conversion not only to Alzheimer’s disease (AD) dementia but also to non-AD dementia, avoiding multiple additional examinations and unnecessary delay in proper clinical management ^25, 32^. Given its high predictive value, ^18^F-FDG PET will likely play a relevant role for patient inclusion in future clinical trials as an accurate tool to select patients at higher risk for short-term conversion to dementia ^34^.

Notably, domain-specific cognitive deficits are known to be associated with network-specific functional alterations, which can be readily detected by ^18^F-FDG PET ^35^. The close relationship between ^18^F-FDG PET and cognitive dysfunctions qualifies this tool as being relevant in the evaluation of treatment outcomes in patients with dementia ^36– 41^. Considering all of the above, we strongly recommend the introduction of ^18^F-FDG PET in future clinical trials, also for the evaluation of therapy outcomes.

To date, a number of international workgroups and consortia have advocated for the relevance of ^18^F-FDG PET in the diagnostic work-up of neurodegenerative diseases ^1– 6, 8, 34^. Nevertheless, a recent Cochrane review concluded that there is not enough evidence supporting the routine clinical use of ^18^F-FDG PET in the diagnosis of dementia in the prodromal phase ^42^. This outcome was likely the consequence of the great methodological heterogeneity across ^18^F-FDG PET literature, including study design, clinical cohorts, and, crucially, data analysis procedures, which are likely to considerably influence ^18^F-FDG PET accuracy, as remarked by a reply from the European Association of Nuclear Medicine ^43^.

The choice of appropriate and validated procedures remains a cardinal issue in ^18^F-FDG PET data analysis. As for clinical diagnostic purposes, various quantitative and semi-quantitative approaches have been developed for single-subject analysis (such as statistical parametric mapping (SPM) ^44^ and Neurostat ^45– 47^) ^12^. SPM is one of the most diffuse methods to statistically analyze voxel-wise ^18^F-FDG PET data ^34^ and its accuracy has been validated in clinical research settings ^25, 26, 30, 33^. A recently developed and validated single-subject SPM procedure takes advantage of an optimized spatial normalization, based on a custom ^18^F-FDG-PET dementia-specific template, and of a high statistical accuracy of the resulting SPM t-maps, based on a large normal dataset for comparisons at the single-subject level ^30, 48^. This ^18^F-FDG-PET SPM procedure allows the identification of disease-specific brain hypometabolism patterns in single cases, and in cross-validation studies for diagnostic accuracy it performs better than visual qualitative assessment of ^18^F-FDG-PET uptake images, clinical assessment of patients, cerebrospinal fluid biomarkers, and amyloid PET ^25, 33^. This method has been validated in clinical research settings both for differential dementia diagnosis ^23, 25, 26, 30^, including atypical parkinsonisms ^26^, and for prognosis in prodromal cases ^25, 30, 32, 33^. We strongly support the implementation of this method not only in academic research but also in routine clinical settings. Other *in vivo* neuroimaging or cerebrospinal fluid biomarkers may also be added in the diagnostic work-up of cases with unclear ^18^F-FDG-PET hypometabolism patterns, likely providing further support to dementia diagnosis.

In the last few years, neurodegenerative conditions have been progressively conceived as diseases of neural networks, which led to the development of multiple statistical multi-variate approaches able to capture their large-scale biological complexity ^49, 50^. ^18^F-FDG PET has been successfully used as a proxy to evaluate metabolic connectivity, assuming that regions whose metabolism is correlated are functionally interconnected ^51^. Of note, distinct patterns of connectivity alterations are associated with different neurodegenerative conditions ^52^. A reduced metabolic connectivity in the hippocampi and in the dorsolateral prefrontal cortex networks was reported in AD ^53^, with an intermediate level of impairment found in MCI ^54^. In contrast, a study on dementia with Lewy bodies (DLB) found altered metabolic connectivity in the occipital cortex, cerebellum, thalamus, and brain stem ^55^. In Parkinson’s disease (PD), an extensive decrease of connectivity in frontal regions, brain stem, and cerebellum was present ^56^. Though compelling, the net majority of these findings are based on group-level analysis, and therefore the single-subject diagnostic value of metabolic connectivity approaches remains to be determined.

## Amyloid PET

The first developed tracer for amyloid imaging was the ^18^F-FDDNP, eventually discarded for its affinity to both amyloid and tau ^57^. Later, the “Pittsburgh compound B” ( ^11^C-PiB) was developed, despite the limitation of the carbon-11 short half-life, requiring an on-site cyclotron and limiting its use in clinical settings. Since 2012, ^18^F-labelled amyloid radiotracers with longer half-life entered clinical and research evaluation in AD ( ^18^F-florbetapir, ^18^F-florbetaben, and ^18^F-flutemetamol) ^58^. Since cortical retention for each ^18^F ligand is highly correlated among tracers ^59^, amyloid-PET data obtained from different tracers may also be compared provided that standardized acquisition procedures ^60^ and specific methods of analysis, such as the recently proposed Centiloid method ^61^, are adopted. Standardization of acquisition procedures and data analysis will certainly improve the incorporation of amyloid-PET biomarkers into the standard diagnostic criteria for AD ^3, 5, 7^. Both ^11^C-PiB and ^18^F-labelled amyloid-PET tracers bind with high affinity to fibrillar amyloid in neuritic plaques ^62– 65^. However, diffuse plaques with low fibrillarity and cerebral amyloid angiopathy (CAA) also contribute to part of tracer binding ^66^.

The large amount of scientific evidence obtained by *in vivo* imaging of brain amyloid burden has challenged the primary role for amyloid in AD pathogenesis ^67, 68^. Still, appropriate use criteria for amyloid-PET imaging stress the high accuracy of amyloid PET in ruling out AD ^69^ and in supporting AD dementia diagnosis, especially in three main clinical populations: (i) subjects with MCI, (ii) patients with suspected AD but atypical presentation or etiologically mixed presentation, and (iii) patients with early-onset dementia ^70, 71^. Of note, in MCI, recent studies have shown that risk to convert to AD is greater in amyloid-positive subjects ^72^ and that the time of conversion from MCI to AD dementia negatively correlates with the annual increase in amyloid deposition rate ^73^. However, since amyloid deposition rate increases rapidly in the prodromal phases of the disease ^74^, in some subjects it might have already reached a plateau in the MCI stage ^22^. For this reason, it has recently been suggested that amyloid rate might not be the best predictor of time to conversion from MCI to AD dementia as compared with neuronal injury biomarkers such as ^18^F-FDG PET ^22^. It should be taken into account that the characteristics of ^18^F-FDG-PET hypometabolic patterns in MCI individuals can indicate the prognostic risk of progression to different dementia conditions (see above) ^23– 25, 32^. In addition, amyloid-PET studies showed MCI patients presenting with wide variations in amyloid burden load ^75– 77^, sometimes showing an intermediate “gray area” burden, challenging attempts to classify it as either positive or negative ^78^.

In patients with dementia, amyloid PET is recognized as a powerful tool to differentiate conditions characterized by a prominent amyloid deposition (that is, dementia due to AD) from those without, such as frontotemporal dementia (FTD) ^79^. Recently, a comprehensive meta-analysis indicated that accuracy of amyloid PET in differential diagnosis of dementia might actually be circumscribed to relatively young demented patients since amyloid positivity dramatically increases with age in cognitively normal individuals (high prevalence after 70 years of age) and in non-AD dementia ^80, 81^. Furthermore, amyloid PET cannot distinguish specific AD syndromes ^82, 83^ and other pathologies characterized by amyloidosis, such as DLB and CAA, and most studies show a non-specific pattern of amyloid burden diffusely distributed throughout the entire cortex across diseases ^84– 86^. Of note, in CAA, ^11^C-PiB was consistently shown to bind to cerebrovascular amyloid plaques, especially in the occipital cortex ^66, 87^. As a consequence, amyloid tracers should be regarded as a general marker of brain amyloidosis and not of specific AD amyloidosis ^12, 66^.

As for amyloid positivity in normal aging, it is currently debated whether cognitively normal subjects with a positive amyloid-PET scan represent prodromal AD cases who will eventually develop AD dementia or rather will remain stable ^9, 67, 78, 88^. Since variations in amyloid deposition rate in normal individuals are subtle (that is, 2–5%) ^89^, a long follow-up might be necessary to observe development of dementia, and up to 19 years might be needed to reach the mean value of amyloid observed in AD ^90^. However, the view that amyloid positivity equals AD dementia diagnosis has been challenged by the high prevalence of amyloid positivity in the elderly (about 44% in nonagenarians) despite normal cognitive function ^80^. Of note, the classification of amyloid-positive or -negative is dependent on the cutoff selected for positivity ^78^, which varies according to the quantification method adopted for tracer binding estimation. In this regard, different quantification methods for amyloid PET have been proposed, including either compartmental model binding tools or reference tissue model-based tools, and various regions of interests are suggested for the latter (for instance, ^91^). A critical issue in amyloid-PET data analysis relates to the definition of the optimal reference region for semi-quantification. Most of the amyloid-PET studies tend to consider the cerebellum, as either a whole or gray matter only, as an optimal reference region, but the pons has been suggested as well ^92^. Particular caution is needed in the case of longitudinal evaluation of amyloid-PET changes, where a direct standardized uptake value ratio (SUVR) semi-quantification could be sensitive to flow changes and produce biased results ^91^. In longitudinal studies, the inclusion of a supratentorial white matter region should be considered for a robust reference ^93^. It is generally accepted that compartmental models provide the most accurate results, and, for routine scans, a simplified reference tissue can be used ^94, 95^. Still, no specific guidelines for amyloid PET have been established yet ^91^. Identification of an optimal normal versus abnormal cutoff with amyloid PET is also crucial in patient selection and inclusion in clinical trials.

Currently, amyloid PET imaging is a crucial requirement for inclusion/exclusion in clinical trials. The majority of clinical trials in AD have focused on amyloid therapies (for example, see references 96– 98), also including serial amyloid-PET scans to evaluate decreases in cerebral amyloid burden ^97, 98^. An open question remains as to which amyloid-PET tracers are the most adequate to evaluate the efficacy of therapeutic interventions. Crucially, the adoption of amyloid-PET imaging for clinical trials has been criticized because currently available amyloid PET tracers measure fibrillary insoluble amyloid burden and are insensitive to toxic soluble amyloid oligomers ^99^, which are much more clinically relevant ^100^. From the methodological standpoint, clinical trials may suffer from the lack of appropriate quantification, usually limited to SUVR semi-quantification (for example, see references 96– 98). Furthermore, the lack of a strong association between amyloid burden and measures of cognition and neurodegeneration ^68^, as shown by several multi-tracer PET studies ^82, 83, 101– 104^, suggests that clinical trials should include other pathological and topographical neurodegeneration biomarkers.

## Tau PET

One of the most recent advances for *in vivo* PET imaging is the evaluation of cerebral tau burden ^105– 108^. Tau protein is physiologically associated with the stabilization, assembly, and functional integrity of microtubules, critical structures for cytoskeletal support and intracellular molecular transport ^109, 110^. Its hyperphosphorylation and accumulation are key pathogenic events in a number of neurodegenerative conditions (that is, tauopathies) and can potentially trigger remarkably different clinical phenotypes and disease courses ^109, 111^. Tau protein can present in six different isoforms, which are grouped on the basis of the number of microtubule-binding domain repeats (that is, three or four (3R/4R)) ^110^. In a normal brain, the ratio of 3R/4R tau is 1:1, but it can change across the different pathologies, such as in the case of progressive supranuclear palsy (PSP) and corticobasal degeneration (CBD), which are 4R-dominant, or in the case of Pick’s disease, which is 3R-dominant ^108, 109^. When agglomerating, hyperphosphorylated tau can additionally assume different conformations, such as paired helical filaments (PHFs), straight filaments, and irregular filaments ^109^. This biological complexity implies a considerably heterogeneous pathological picture which historically hampered the development of selective tau radioligands suitable for *in vivo* PET imaging ^106^.

Notwithstanding tau biological complexity, the synthesis of some radioligands is now available and is rapidly entering into extensive research use and possibly for a potential validation in clinical practice ^108^. To date, three broad groups of radioligands are under extensive evaluation: the ^18^F-THK5351, ^18^F-THK5117, or ^18^F-THK5105 ^112– 114^; the ^18^F-T807 (also known as ^18^F-AV1451 or ^18^F-Flortaucipir) ^115^; and the ^11^C-PBB3 ^116^.

The first tau PET imaging results in the AD spectrum have provided compelling evidence for a tight relationship between tau burden and synaptic dysfunction, gray matter atrophy, and cognitive deficits ^117– 120^, confirming previous post-mortem evidence ^121, 122^. Early reports additionally suggested that tau PET was able to recapitulate the neuropathological Braak staging, suggesting that it could be a valuable tool for the *in vivo* staging of AD pathology progression ^120, 123^. Multiple reports showed a cross-sectional association between worsening of cognitive impairment and increasing cortical tau-PET binding, from normal cognition to MCI and AD dementia stages ^117, 118, 120^. Of note, the correspondence between tau accumulation, neurodegeneration, and clinical manifestations stands in stark contrast with amyloid-PET evidence, which is not associated with specific patterns of neurodegeneration or cognitive impairment (see “Amyloid PET” section above). The topographical specificity of tau-PET uptake distribution especially emerged for the AD variants, such as posterior cortical atrophy and the logopenic variant of primary progressive aphasia, which are known to be associated with phenotype-specific patterns of neurodegeneration and cognitive deficits ^124^. These studies showed a consistent spatial correspondence between *in vivo* tau burden, neurodegeneration, and clinical syndromes, at group and single-subject levels ^83, 119, 125– 127^.

The introduction of tau-PET techniques is reshaping the AD research field, allowing a more targeted evaluation of the original amyloid cascade hypothesis, which remains highly controversial (see “Amyloid PET” section above and ^67, 128^). Several studies specifically focused on the relationships between tau and amyloid accumulation in AD and in healthy aging ^119, 120, 129– 133^. The most consistent and compelling observation regards the variable patterns of tau deposition in subjects with or without a considerable cerebral amyloid burden ^108^. Medial temporal lobe (MTL) tau accumulation has been associated with an age-related process independent from amyloid burden ^133^, whereas tau spreading outside the MTL is almost invariantly associated with an amyloid-positive status ^132– 135^.

While providing a wealth of evidence with critical implications for disease tracking and monitoring of AD interventions, the above-mentioned studies have also highlighted several areas of criticisms which are in need of further consideration ^136^. Of note, the selectivity of the current tau-PET radioligands for non-AD tauopathies is not well understood. Previous autoradiographical studies on the most commonly adopted tau-PET compounds, such as the ^18^F-AV1451, have shown high affinity for the AD tauopathy (that is, for intracellular neurofibrillary tangles composed of PHFs, with an equal 3R/4R tau ratio) ^137, 138^. The same, however, was not observed for the 4R tau aggregates typical of primary tauopathies, such as PSP or CBD, where post-mortem results are more heterogeneous and overall present less robust staining ^137– 142^. Another area of concern regards consistent non-specific tau-PET binding in subcortical structures, especially in the striatum and in the choroid plexus, in healthy controls, suggesting that the currently available tau tracers could present off-target binding, such as to neuromelanin ^137, 138^. The possibility that tau-PET ligands present non-specific binding is supported by a recent study showing that selegiline, a monoamine oxidase-B (MAO-B) inhibitor, significantly reduces brain ^18^F-THK5351 uptake ^143^. Additionally, it has been suggested that AV1451 could present non-specific binding to MAO-A as well ^144^, even if there are opposite results ^145^. Another issue in tau-PET quantification is represented by the time window adopted for SUVR semi-quantification. As for ^18^F-AV1451, the 80- to 100-minute SUVR, commonly adopted in previous research studies ^136^, might be not optimal given the evidence for a further 30% increase of SUVR values up to 180 minutes from injection ^146^. The longer uptake time window of this tracer should be taken into account when such semi-quantifications are adopted ^146^. Given these premises, tau-PET diagnostic value in AD, and especially in non-AD tauopathies, is in need of further evaluation.

## Neuroinflammation PET

*In vivo* imaging of neuroinflammation responses has recently gained particular interest in clinical neuroscience research ^147, 148^, and neuroinflammation has been recognized as a key player in the course of neurodegeneration ^149^. The currently available PET molecular imaging techniques allow the measurement of neuroinflammation through imaging of both astrocytes and microglia activation ^147^. Astrocytosis can be uniquely measured through PET and the ^11^C-deuterium-L-deprenyl ligand ( ^11^C-DED), which is an irreversible inhibitor of the MAO-B enzyme, over-expressed during astrocyte activation ^150^.

The great majority of research has otherwise focused on PET imaging of microglia activation, and many radioligands have been synthetized, the great majority of which target the over-expression of the 18-kDa translocator protein (TSPO). TSPO is an outer mitochondrial membrane protein, expressed mostly by microglia and, to a lesser extent, by astrocytes ^151, 152^. Its levels in the central nervous system in healthy conditions are generally low, whereas their over-expression in the disease state has been well documented ^152^.

Among the others, ^11^C-(R)-PK11195 is the first and prototypical TSPO radioligand ^153^ and has been widely adopted in multiple neurodegenerative conditions ^147, 154^. A second generation of radioligands has been subsequently introduced, mainly to overcome some of the ^11^C-(R)-PK11195 limitations, such as limited availability of ^11^C ligands in clinical settings and low signal-to-noise ratio ^155^. These second-generation ligands come with both ^11^C or ^18^F isotopes, such as ^11^C-PBR28 and ^18^F-DPA714, and overall display higher binding affinities ^155^. A few years ago, however, it was shown that a single-nucleotide polymorphism (that is, rs6971) in the TSPO gene can considerably influence the uptake of the second-generation tracers, making genetic testing mandatory ^156, 157^. While a third generation of fluorinated radioligands is currently under evaluation ^158^, the ^11^C-(R)-PK11195 remains the best validated and diffusely adopted in human studies, not requiring TSPO genotype evaluation.

Of note, quantification of TSPO PET can be particularly challenging ^159^ given the intrinsic characteristics of the TSPO protein ^159^. For instance, TSPO can be remarkably expressed in the endothelium, and it is heterogeneously distributed across the brain, hindering the definition of an anatomically defined reference region ^159^. To overcome the latter limit, several advanced clustering algorithms have been developed to iteratively select groups of voxels sharing specific time activity curves (that is, resembling temporal delivery of the tracer in gray matter without specific binding) ^160– 162^. The complexity of TSPO-PET quantification and the use of radioligands with different properties, together with the characteristics of TSPO distribution, likely contributed to the pronounced heterogeneity of the reported findings in the literature. A representative example comes from TSPO-PET imaging of AD ^154, 155, 163^. Previous studies have shown significant microglia activation in key AD signature regions, such as entorhinal and temporo-parietal areas, whereas some have reported negative results ^155^. As for relation to disease phase, some studies described more significant microgliosis ^164^ and astrogliosis ^165^ in prodromal/preclinical rather than AD dementia phases whereas others showed the opposite ^166^. Of note, previous ^11^C-DED-PET studies have accordingly shown more significant astrocytosis in prodromal and preclinical genetic AD subjects when compared with subjects in later disease phases ^165, 167^. Diverging evidence also exists regarding the association between microglia activation and amyloid burden ^168– 170^. Several recent studies have provided novel evidence for longitudinal associations between neuroinflammation, neurodegeneration, and pathology accumulation in AD ^165, 171, 172^. An increase of microglia activation in AD was longitudinally associated with amyloid accumulation and reductions of brain glucose metabolism ^171^. Another recent study introduced a possible “two-peaks” model of microglia activation in AD ^172^ with microgliosis first peaking at the prodromal MCI stage, afterwards declining approaching AD dementia transition, and then increasing again during the final disease stages ^172^. As for ^11^C-DED PET, a recent longitudinal study on carriers of autosomal dominant AD mutations also indicated that the highest detected astrocyte activations were present at the asymptomatic stage, progressively decreasing approaching clinical onset ^165^. Of note, amyloid accumulation followed an opposite trend ^165^.

As for other neurodegenerative conditions, TSPO PET has been successfully applied in synucleinopathies, such as in DLB, PD, and PD with dementia ^173– 175^; in FTD ^176^; in atypical parkinsonisms such as multiple system atrophy (MSA), CBD, and PSP ^177– 179^; and in prion diseases, such as Creutzfeldt-Jakob disease ^180^.

Crucially, it remains to be understood whether this local immune response is thoroughly beneficial or rather can promote neurodegeneration ^149^. This is particularly true in relation to microglia activation, which is a complex and dynamic process ^181^. Understanding whether and how microglia is reacting within a disease course may provide important insights into disease pathogenesis and have remarkable implications for future clinical trials. PET imaging of neuroinflammation provides the unique chance of evaluating, *in vivo*, the reactivity of microglia cells, but also astrocytes by means of ^11^C-deprenyl PET, to either neuronal damage or pathology accumulation, with remarkable implications for research and possibly future clinical settings ^154, 163^.

Further validation of TSPO-PET imaging is needed to translate its application to clinical practice. Notwithstanding the broad efforts, the greatest challenge is still represented by the lack of a standardized methodology, which hampers the reproducibility and the biological interpretation of the findings. Additionally, the debate on the actual physiological role of the TSPO protein is yet to be solved ^182, 183^. One of the most remarkable future applications of neuroinflammation PET is the outcome evaluation of pharmacological interventions. Some studies have already used TSPO PET to monitor the anti-inflammatory or immunomodulatory therapies in PD ^184^, MSA ^185^, and multiple sclerosis ^186, 187^.

Several non-TSPO new targets to measure microglia activation with PET-based techniques, including but not limited to purinergic and cannabinoid receptors, are currently under evaluation ^188^. The development of these new radioligands not only is linked to the attempt of overcoming TSPO intrinsic limits (see above) but also aims at the detection of specific microglial functional phenotypes ^188^. In neurodegenerative conditions, microglia can acquire very diverse functional phenotypes based upon several factors ^189^; in this direction, the development of PET radioligands with particular affinity for specific microglial polarizations (for instance, homeostatic versus neurotoxic) would be of utmost importance. Preliminary data are available for PET imaging of the cannabinoid receptor type 2 (CB2R), which is over-expressed by microglial cells during activation and has been shown to promote neuroprotection ^190^. The CB2R PET radioligand, ^11^C-NE40, has been recently used in patients with AD ^191^. The development of PET tracers addressing phenotype-specific microglia activation will hopefully allow novel insights into how neuroinflammation responses could be contributing to neurodegeneration.

## Conclusions

Recent studies have progressively highlighted how the same pathology can trigger very diverse functional phenotypes. Given the recent advances in neuroimaging techniques, it is likely that the multi-modal integration of pathological and functional biomarkers will be the key proxy to the most accurate identification of both underlying pathology and phenotypic syndrome, leading to the tailoring of the most appropriate care plan and prognosis. The increasing availability of PET *in vivo* pathology markers will likely favor the implementation of a spectrum-based research framework.

Although the clinical usefulness of amyloid PET is recognized, it is particularly recommended to specific clinical sub-populations, such as early and atypical clinical presentations. The novel tau tracers are promising, given their tight relationship with neurodegeneration, but the lack of affinity for different tau isoforms and the evidence for non-specific bindings shown by several of these radioligands call for the development of novel compounds overcoming these limitations. In this context, ^18^F-FDG PET provides a well-validated key value to dementia diagnosis and prognosis and should be considered as one of the most valuable tools for monitoring neurodegenerative disease status and progression and also for selecting candidates for clinical trials and evaluating treatment response in both AD and non-AD pathologies.

## Abbreviations

^11^C-DED, ^11^C-deuterium-L-deprenyl; ^11^C-PiB, carbon-11 Pittsburgh compound B; ^18^F-FDG, fluorine-18 fluorodeoxyglucose; AD, Alzheimer’s disease; CAA, cerebral amyloid angiopathy; CB2R, cannabinoid receptor type 2; CBD, corticobasal degeneration; DLB, dementia with Lewy bodies; FTD, frontotemporal dementia; MAO, monoamine oxidase; MCI, mild cognitive impairment; MSA, multiple system atrophy; MTL, medial temporal lobe; PD, Parkinson’s disease; PET, positron emission tomography; PHF, paired helical filament; PSP, progressive supranuclear palsy; SPM, statistical parametric mapping; SUVR, standardized uptake value ratio; TSPO, translocator protein.
